# Commentary: Outbreak of Chikungunya in Pakistan

**DOI:** 10.3389/fpubh.2017.00261

**Published:** 2017-09-28

**Authors:** Tauqeer Hussain Mallhi, Yusra Habib Khan, Amer Hayat Khan, Nida Tanveer, Omaid Hayat Khan, Raja Ahsan Aftab

**Affiliations:** ^1^Discipline of Clinical Pharmacy, School of Pharmaceutical Sciences, Universiti Sains Malaysia, Penang, Malaysia; ^2^Punjab Medical College, Faisalabad, Pakistan; ^3^Department of Pharmacy, The University of Lahore, Lahore, Pakistan

**Keywords:** chikungunya, Pakistan, vector-borne diseases, India, outbreak

Rauf et al. in their recent correspondence in “*Lancet Infectious Diseases*” reported the first chikungunya outbreak in Karachi, Pakistan with 30,000 suspected and 4,000 confirmed cases (1). However, these estimates have been denied in a subsequent report by the National Institute of Health (NIH) indicating 818 suspected and 82 laboratory-confirmed cases of chikungunya (2). Rauf and colleagues have highlighted warm climate and wretched sanitary conditions as contributing factors of current outbreak and urge national and international health-organizations to address these momentous issues (1). We agree that climatic features and sanitation issues potentially lead to vector proliferation and the importance of these concerns cannot be disregarded. However, we felt inclined to share our point of view about the recent outbreak of chikungunya in Pakistan. We believe that there are some more important factors that should be considered as causes of this outbreak and must be addressed by the Government of Pakistan in haste to quell the further disease spillover. One of these factors is unchecked cross-border movements between Pakistan and India.

It is pertinent to mention that Pakistan experienced a chikungunya outbreak after few months of its outbreak in India. Although NIH had warned Pakistan about the potential risks of chikungunya transmission from India and urged the Government to keep vigil on cross-border traveling, but no preventive measures were taken at airports, railway stations, and the Indo–Pak borders (3). Pakistan shares 2,912-km eastern border with India and hundreds of people travel between two countries on daily basis by several routes such as train, bus, and plane. However, there are no health screening facilities at airports and border-crossing points of both countries that pose a substantial risk of spillover infection across borders (4). Intensified trade and travel render political borders irrelevant and create further possibilities of global disease transmission. There are numerous examples of spread of exotic pathogens to new geographic locations by cross-border movements including malaria, dengue, Zika, West Nile, and Crimean-*Congo* hemorrhagic fever (CCHF). This process is facilitated when the environmental conditions across borders share common characteristics (5). Pakistan and India have similar climatic conditions that prodigiously support vector proliferation. As compared to Pakistan, the range and burden of vector-borne diseases (VBDs) are enormous in India including severe acute respiratory syndrome contagion in 2002–2003, influenza A virus epidemic of 2006 (avian influenza), 2007 equine influenza, swine flu pandemic outbreaks in 2009 and 2014 (4). Moreover, VBDs continue to rise in India, with a recent surge of chikungunya and dengue cases in 2016 (6). In the light of these facts, we believe that probability of chikungunya virus transmission from India to Pakistan should not be ignored.

Every year, thousands of Indians (Sikh community) visit Pakistan to celebrate the birth anniversary of their founder at Gurdwara temple (his birthplace), and most of them enter in Pakistan through train or bus service. With the 500th anniversary of the temple’s founding coming up in 2022, many more Sikhs are likely to visit Pakistan (7). In addition, bilateral trade ties between two countries also increase the risk of vector transmission across borders. We strongly believe that the infectious diseases can easily spread with infected travelers or trade commodities entering the Pakistan. Hence, there is a dire need that the Pakistani government should develop strategies to screen people by establishing the health units at airports and border-crossing points. It is essential to implement entry and exit screening procedures at airports within the context of the International Health Regulations to slow down the spread of infection, especially during the early phases of a pandemic event. Moreover, collaborative efforts among two countries on vector surveillance and control could go a long way to combating VBDs in both regions.

Vector-borne diseases account for over 17% of all infectious diseases, causing more than one million deaths annually (8). Chikungunya virus also circulates in Pakistan (1), and we believe that factors including climate vagaries, poor sanitation, unchecked border crossings, unplanned urbanization, and changes in agriculture practices contribute to the recent chikungunya outbreak in the country. Moreover, the year 2016 portrayed a dire situation for infectious diseases in Pakistan a dengue epidemic, 19 deaths attributed to CCHF, and first chikungunya outbreak (9, 10). Government of Pakistan should pay more attention to vector control to interrupt transmission of VBDs. In addition to border screening, aggressive maneuvers on environmentally safe insecticides, research on alternative approaches (such as biological control), development and implementation of integrated prevention strategies, appropriate human resources to develop and implement sustainable prevention programs, and adequate training of personnel are required to reverse the current trend of VBDs in Pakistan.

## Author Contributions

All the authors equally contributed to the manuscript. YK, RA, and NT searched for literature to support main content of the manuscript. TM and AK drafted the manuscript. TM and OK revised manuscript and gave final approval to submit this manuscript. However, all the authors agreed to submit this manuscript.

## Conflict of Interest Statement

The authors declare that the research was conducted in the absence of any commercial or financial relationships that could be construed as a potential conflict of interest.
